# The contribution of employer characteristics to continued employment of employees with residual work capacity: evidence from register data in The Netherlands

**DOI:** 10.5271/sjweh.3961

**Published:** 2021-08-31

**Authors:** Raun van Ooijen, Pierre WC Koning, Cécile RL Boot, Sandra Brouwer

**Affiliations:** Department of Health Sciences, Community and Occupational Medicine, University Medical Center Groningen, University of Groningen, The Netherlands; Department of Economics, VU University Amsterdam, The Netherlands & Department of Economics, Leiden University, Leiden, The Netherlands; Department of Public and Occupational Health, Amsterdam Public Health research institute, Amsterdam UMC, VU University Amsterdam, The Netherlands

**Keywords:** Key terms disability, disability-related policy, disability-related practice, employee characteristic, employer characteristics, partial disability, work participation

## Abstract

**Objectives:**

This study aimed to examine the contribution of employer characteristics to continued employment of employees with residual work capacity. Moreover, we examined whether the contribution of employer characteristics differs across types of employers and employees’ types of diseases.

**Methods:**

Register data on disability assessments and employment status of N=84 394 long-term sick-listed employees with residual work capacity were obtained from the Dutch Employee Insurance Agency between 2010 and 2017. The dependent variable was continued employment four months after the assessment. We linked employees to their (former) employer to measure sector, firm size, and workforce composition. The average employment outcome of all employees assessed in the same firm and year served as a proxy measure for the extent of implemented disability-related policies and practices. Using multilevel multiple regression analysis, we compared the relative contribution of employer characteristics with employees’ characteristics.

**Results:**

Employer characteristics accounted for 10% of the variability in employment outcomes. In comparison, employees’ socio-demographic and disease characteristics accounted for 13% of the variability. The prevalence of continued employment was lowest in smaller firms and construction and low-wage service-orientated sectors. Furthermore, there were sizeable differences in employment outcomes between similar employers in terms of size, sector and workforce-composition, particularly between larger firms and among employees with mental or musculoskeletal disorders compared to other diseases.

**Conclusions:**

This study shows substantial differences between employers in facilitating continued employment of employees with residual work capacity. Encouraging firms to invest more in disability-related policies and practices may result in better employment opportunities for these employees.

Over the last few decades, many industrialized countries have reformed their disability insurance programs to reduce inflow into long-term disability, particularly by tightening eligibility criteria and incentivizing employers to encourage work participation of long-term sick-listed employees who have residual work capacity (1–3). While these reforms successfully reduced disability inflow, the probability of continuing work after the approval of disability benefits remains low in OECD countries (4). In this context, the employers’ effort to not only invest in the reintegration of long-term sick-listed employees but also to support them after they applied for disability benefits seems a critical factor for continued employment of employees with residual work capacity.

Previous research examined the importance of several employer-related characteristics for return to work of sick-listed employees with chronic diseases, both at the workplace and organizational level. At the workplace level, particularly work accommodations – such as the provision of modified or part-time work – were found to be associated with work resumption (5–8). For social or emotional support by the supervisor, the results are mixed: several studies found significant results (9–13), but others found insignificant results (14–19). At the organizational level, both disability-related policies and practices (16, 20) and the organizational structure of a firm in terms of size, sector, and workforce-composition (eg, age and gender composition) were found to be associated with work resumption (21). Studies on the sector of employment did, however, not reveal any clear pattern (21–26). For firm size, the pattern is more consistent: most studies found that larger companies are more successful in retaining sick-listed employees (6, 21, 26–28) or found no association (16, 22, 23, 25). Only a few studies found a negative association (15, 24).

Whereas the association between various employer-related characteristics and return to work of employees with chronic diseases is well described, there is limited understanding of the employer’s contribution to continued employment of employees with residual work capacity. Most studies focused on the period of sick leave instead of the period after the disability claim assessment. For the design and implementation of disability schemes and return-to-work policies for this disadvantaged group of employees, we therefore need to gain more insight into the importance of employer characteristics for continued paid employment of employees with residual work capacity after applying for disability benefits, either at their current employer or in a new, more appropriate job.

In The Netherlands, employers are financially responsible for employees with an employment contract, during the period of both sick leave and after the disability assessment when they receive disability benefits, and employers pay experience-rated disability benefit premiums. Employers faced limited financial incentives to organize return to work for workers without an employment contract (ie, a flexible or expired temporary contract) up to 2017.

In this context, this study aimed to examine the contribution of employer characteristics to continued employment of long-term sick-listed employees with residual work capacity. We considered several employer characteristics: sector, firm size, workforce-composition, and a within employer effect that proxies the extent of implemented disability-related policies and practices. In particular, we proxy the employer’s performance in organizing continued employment by the share of all of its employees with residual work capacity that continue working after the disability assessment. This presupposes that effective disability-related policies and practices are reflected by the likelihood of work continuation of all employees with residual work capacity. In addition, we compared the contribution of employer characteristics to employees’ socio-demographic and disease characteristics. Furthermore, we evaluated whether employers’ contribution through its policies and practices differs across types of employers (sector and firm size), and employees’ types of diseases. Finally, we conducted a sensitivity analysis to study whether employers’ contribution differs between employees with or without an employment contract before the disability assessment. The idea behind this analysis was that there should be no employer effect on disabled employees’ employment outcomes without employers being responsible for them at the moment of the disability assessment.

## Methods

### Institutional setting

In The Netherlands, social insurance legislation (Work and Income Act; WIA) allows employees to apply for a disability benefit after two years of sick leave (29). Individuals may receive disability benefits for a disease or handicap due to either occupational or non-occupational causes. After a medical disability assessment by an insurance physician and assessment of earning capacity by a labor expert (of the Dutch Employee Insurance Agency; UWV), individuals can either have a full and permanent work disability, a non-permanent but full work disability, or a partial work disability. Individuals in the latter group have residual earnings capacity. Individuals with residual capacity are incentivized to continue in paid (part-time) employment at their current employer or enroll in a new, more appropriate (part-time) job. Individuals should have <65% residual capacity to receive a disability benefit. Individuals disabled since childhood are not entitled to a WIA-disability benefit. Instead, they can apply for a WAJONG-disability benefit when they turn 18 years (Disablement Assistance Act for Handicapped Young Persons).

### Data sets

We used register data on disability assessments from the Claim Assessment and Monitoring System enriched with earning records to measure employment status from the Dutch Employee Insurance Agency (UWV) from January 2010 to April 2017. The earnings records contain employer identifiers that allowed us to link employees with remaining wok capacity to their (former) primary employer and assessed and non-assessed coworkers. For each disabled employee, we observed an extensive set of characteristics that are informative on the type of employer, including sector, firm size, and workforce-composition. Moreover, the linked data provided information on the employment outcomes of all employees in the same company that applied for disability benefits after a period of sick leave. For all non-assessed coworkers, which, as a consequence, are not registered in the Claim Assessment and Monitoring System, we obtained demographic information on their year of birth and gender from the earnings records. Demographic information of all employees in a company was used to construct measures of firms’ workforce composition.

### Inclusion and exclusion criteria

**Figure 1:**
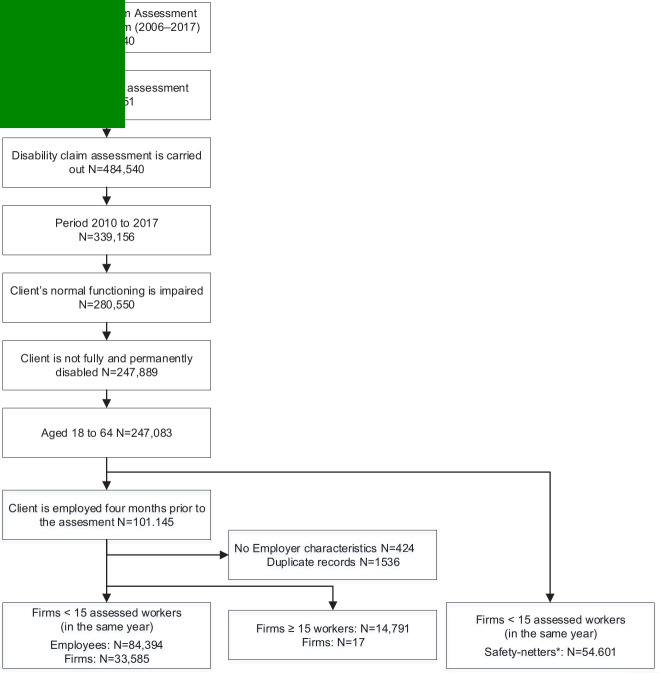
Flowchart of the study population. *Safety-netters are workers with disabilities that had no employment contract four months before the disability assessment. For this group of workers, employers have less incentives to facilitate continued employment.

Figure 1 shows a flowchart of the selected study population from the enriched register data. Our sample included employees aged 18–64 years who applied for public disability benefits after a two-year waiting period of sick leave. Since the earnings records are available as of 2008 and we condition our sample on being employed two years before the disability assessment, our sample effectively covers the years 2010–2017. We excluded persons who were either fully and permanently disabled or able to fully function in their current job, according to the professional opinion of the insurance physician. In this way, we restricted our sample to persons that were considered to be able to continue working after the assessment, either partially or with work adjustments. We refer to these persons as employees with residual work capacity.

The sample was further restricted to employees who had an employment contract four months before the disability assessment with the same employer as to when the period of sick leave started. Employers are financially responsible for workers who were still employed at the moment of the disability claim assessment, during both the period of sick leave and after the disability assessment when they receive disability benefits, and employers pay experience-rated disability benefits premiums. As such, we excluded workers who were not employed four months before the disability assessment. In our sample period, employers faced limited financial incentives to organize return to work for these temporary and flexible workers. As from 2017, employers also face experience-rated disability benefit premiums for these workers.

Finally, we dropped employees with missing information on the characteristics of their employer. For similar reasons, some duplicate records were dropped from the sample. In addition, we selected a subsample that excluded 17 large firms with ≥15 assessed employees in the same year to reduce the influence of a few large firms with a vast number of assessed employees. For our main analysis, this left us with a sample of 84 394 employees with residual work capacity at 33 585 employers.

### Dependent variable

The dependent variable was the employment status four months after the disability assessment. We distinguished between two mutually exclusive employment outcomes: working in a paid job for the same employer or another employer (referred to as continued employment) and not working in a paid job. We considered an employee’s employment status four months after the disability assessment because it could take some time before employment transitions, such as the termination of employment, are administered in the earnings records.

### Independent variables

#### Employer characteristics

Employer characteristics were measured at the year-end of an employees’ disability assessment and are the same for all employees in a particular firm that year. We used register data on the employer level containing the following variables: sector, firm size, and workforce composition. The sector is based on the International Standard Industrial Classifications (ISIC). We distinguished between 12 main sectors. Firm size was categorized into eight groups to address a potential non-linear relationship. By aggregating employee characteristics on the employer level, we created several variables measuring the workforce-composition: the median wage rate, the fraction of female employees, the fraction of employees with a fixed-term contract, and the fraction of employees aged ≥55 years. The workforce-composition variables were all categorized into quartiles.

There are no variables in the register data that are informative on the employer’s disability-related policies and practices. An overall measure that encompassed these policies and practices is therefore constructed. We proxied the extent of implemented policies and practices with the fraction (values 0–1) of disabled coworkers assessed in the same year that continue working after the disability claim assessment. The intuition behind this proxy variable is that the fraction of all employees assessed for disability benefits within the same firm that successfully continue employment reflects the extent to which an employer successfully contributes to the continued employment of all its employees. In turn, this success will depend on the employer’s disability-related policies and practices.

#### Employee characteristics

The employee characteristics are described in the supplementary material (www.sjweh.fi/article/3961), Appendix A.

### Statistical analysis

We used a multilevel linear regression model with a binary dependent variable (also called a linear probability model) to determine how much of the variation in employment outcomes four months after the disability assessment was accounted for by employer characteristics and employee characteristics. The advantage of using a linear probability model with a normal error distribution instead of a logistic model is that it allows for our variance decompositions (30). We are aware that the linear probability model may give predicted values outside the unit (0–1) interval. However, almost all predicted probabilities were inside the unit interval. In our model, the dependent variable takes the value 1 when an employee remains employed four months after the disability assessment, and 0 otherwise. The employment outcomes of 33 585 employers were the level one units, and the 84 394 assessed employees the level two units. On average, per employer, 3.1 employees were assessed in the same year [standard deviation (SD) 3.1]. About half of the employers (50.9%) have one assessed employee, 13.4% two employees, 7.5% three employees, 5.6% four employees, reducing to 1.2% for fourteen employees.

We first estimated a linear probability model of employment outcomes four months after the disability assessment as a function of employer- and employee-level characteristics (model 1) and an employer effect measured by the fraction of disabled coworkers (assessed in the same year) that continue working after the disability assessment (model 2). The employer effect proxies employer-specific disability-related policies and practices as it reflects the extent to which an employer successfully contributes to the continued employment of all its employees. We analyze it by a 10% increase in the fraction of coworkers that continue working. To estimate the coefficient of the employer effect, the sample was limited to employers with at least two assessed employees in the same year. The remaining coefficients were derived for the full sample of employees by including an indicator variable where 1=employers with only one assessed employee in a given year and 0=otherwise. As other employer- and employee-level characteristics could influence employment outcomes, we include them in model 2 as well. In all models, we also accounted for time-varying period effects by including year-dummies.

To determine how much of the associations are accounted for by specific employer- and employee characteristics, we then estimated eight sequential models: four on the employer-level (sector, firm size, workforce-composition, and an employer effect measuring disability-related policies and practices), three on the employee-level (demographic, socioeconomic and disease characteristics), and one for period effects. In sequential analysis, the results can be sensitive to the order in which groups of characteristics are excluded from the model (31). To address such problems, we proceeded in two steps. First, for all possible combinations in which groups of characteristics can be excluded from the model, we estimated the contribution of these groups of characteristics to the total variance in employment outcomes. Next, we took the average value (31, 32).

The employer effect cannot be directly derived from model 2. It depends non-linearly on the total number of assessed coworkers in a company in a given year, as we set out in supplementary Appendix B. We obtained our measure of interest by estimating separate regression models (of model 2) for firms with the same number of assessed coworkers in a given year and aggregating the derived estimates using inverse-variance weighting. In addition, to determine whether the employer effect differs across employers and employees, the variables sector, firm size, and disease groups were assessed as moderators. Interaction terms with the indicator variables of the sector of employment, firm size groups, and employee’s type of disease groups were therefore added to the model.

Finally, we conducted a sensitivity analysis to examine whether the employer effect was different for employees without an employment contract before the disability assessment. This analysis should yield insignificant effects of the employer effect. For this falsification test, we extended our sample with temporary and flexible workers and interacted the employer effect with an indicator variable of having an employment-contract before the disability assessment (model 3).

## Results

### Sample descriptives

Table 1 presents descriptive statistics for our sample of employees with residual work capacity. It showed that 50.6% of the employees continued in paid employment four months after the disability assessment. Most of them (87.6%) remained at their current employer. The mean fraction of coworkers who continue working after the disability assessment (56%) is slightly above the fraction for the employee which might be related to the larger average firm size for those who have disabled coworkers assessed in a given year. The mean age of the sample was 47.7 (SD 10.0) years, and more than half (54.8%) was female. The two most frequently diagnosed type of disease groups were musculoskeletal (31.6%) and mental disorders (24.0%). For mental work incapacity, the mean value of 0.05 indicates that few employees have severe mental work incapacities. That is because our sample contains employees with residual work capacity. For physical work incapacity, the mean value is about three times as large (0.18), indicating that a more substantial group has serious physical health problems. As expected, the coworker averages for work incapacity have similar mean values as for employees themselves.

**Table 1:** Characteristics of the study population (N=84 394). Fraction and percentage employed four months after the assessment (mean=50.6%). [SD=standard deviation.]

Employer characteristics	Fraction %	Employed %	Mean	SD
Sector				
Agriculture	2.0	45.6		
Construction	4.3	40.1		
Manufacturing	17.1	49.0		
Wholesale and retail	13.9	41.9		
Transportation	5.2	43.5		
Recreation	3.2	35.7		
Financial and insurance	1.3	65.9		
Professional activities	8.7	52.2		
Support services	5.7	31.1		
Public administration	8.2	74.5		
Education	7.8	73.1		
Health	22.7	50.2		
Firm size				
1–9	10.9	37.8		
10–49	19.2	41.9		
50–99	8.4	45.3		
100–249	12.3	50.5		
250–499	9.9	55.3		
500–999	11.2	56.0		
1000–4999	25.4	59.0		
≥5000	2.8	61.0		
Workplace earnings (quartile)				
Lowest	24.2	39.2		
2^nd^	26.4	45.4		
3^rd^	26.1	56.7		
Highest	23.3	61.4		
Fraction woman (quartile)				
Lowest	25.8	48.4		
2^nd^	24.2	52.6		
3^rd^	22.2	55.2		
Highest	27.8	47.1		
Fraction fixed contract (quartile)				
Lowest	25.8	42.6		
2^nd^	26.1	53.6		
3^rd^	23.8	56.9		
Highest	24.3	49.6		
Fraction age>55 (quartile)				
Lowest	27.3	43.3		
2^nd^	24.4	51.5		
3^rd^	24.4	53.7		
Highest	23.8	54.7		
Coworker averages (0–1)				
Continued employment			0.56	0.37
Mental work incapacity			0.05	0.05
Physical work incapacity			0.18	0.10
Age (years)			47.7	10.0
18–29	5.3	49.7		
30–34	7.8	48.4		
35–39	9.9	48.8		
40–44	12.5	49.8		
45–49	15.4	50.2		
50–54	18.7	51.5		
55–59	19.2	53.0		
60–64	11.2	49.6		
Gender				
Men	45.2	51.4		
Women	54.8	49.9		
Education				
Primary	16.8	34.3		
Lower secondary	27.6	42.5		
Upper secondary	28.8	52.0		
Tertiary	14.2	64.6		
Missing	12.6	71.0		
Earnings (quartile)				
Lowest	24.4	37.1		
2^nd^	25.0	45.5		
3^rd^	25.9	53.4		
Highest	24.7	66.1		
Primary diagnosis				
Cancer	8.0	68.4		
Mental	24.0	44.9		
Nervous	5.0	60.4		
Circulatory	7.2	59.2		
Musculoskeletal	31.6	43.9		
Injury	7.7	52.9		
Other	16.4	55.2		
Comorbidity				
No	56.9	54.8		
Yes	43.1	45.0		
Work incapacity (0–1)				
Mental			0.05	0.07
Physical			0.18	0.13

### Multilevel analyses

Table 2, column 1, reports the results from the linear probability model 1. In total, 19.9% of the variability in continued employment was explained by included variables: sector, firm size, workforce composition, and employees’ socio-demographic and disease characteristics. (The estimated coefficients for the included employee characteristics are presented in supplementary table S1.) Continued employment was positively associated with firm size: employers with >5000 employees showed a 12% higher employment rate than those that employed <10 employees. Employees in public organizations (education and public administration) had the highest employment rate: about 14.3% and 20.7% higher than employees in manufacturing (reference group). Employees in the support services sector had the lowest employment rate: 7.8% lower than those in manufacturing.

**Table 2:** Multiple linear regression analysis with continued employment four months after the assessment as dependent variable [β=coefficients; SE=standard error].

Employer characteristics ^a^	Model 1 (N=84 394) R^2^=0.199			Model 2 (N=84 394) R^2^=0.205			Model 3 (N=134 101) R^2^=0.303		
		
	β	SE	P-value	β	SE	P-value	β	SE	P-value
Sector									
Agriculture	0.017	0.012	0.15	0.017	0.012	0.16	0.017	0.008	0.05
Construction	-0.077	0.009	0.0	-0.071	0.009	0.0	-0.041	0.006	0.0
Manufacturing	Reference			Reference			Reference		
Wholesale and retail	-0.015	0.006	0.01	-0.013	0.006	0.04	-0.002	0.004	0.60
Transportation	-0.047	0.008	0.0	-0.046	0.008	0.0	-0.018	0.005	0.0
Recreation	-0.040	0.010	0.0	-0.038	0.010	0.0	-0.006	0.006	0.32
Financial and insurance	0.050	0.015	0.0	0.037	0.015	0.01	0.021	0.011	0.06
Professional activities	0.001	0.007	0.84	0.000	0.007	0.99	0.003	0.005	0.53
Support services	-0.078	0.008	0.0	-0.067	0.008	0.0	-0.013	0.005	0.01
Public administration	0.207	0.008	0.0	0.175	0.008	0.0	0.152	0.006	0.0
Education	0.143	0.009	0.0	0.118	0.009	0.0	0.129	0.006	0.0
Health	0.007	0.007	0.31	0.004	0.007	0.61	0.020	0.005	0.0
Firm size									
1–9	Reference			Reference			Reference		
10–49	0.018	0.006	0.0	0.022	0.006	0.0	0.014	0.004	0.0
50–99	0.040	0.007	0.0	0.049	0.007	0.0	0.026	0.005	0.0
100–249	0.061	0.007	0.0	0.074	0.007	0.0	0.041	0.005	0.0
250–499	0.069	0.007	0.0	0.084	0.008	0.0	0.051	0.005	0.0
500–999	0.093	0.007	0.0	0.106	0.008	0.0	0.063	0.006	0.0
1000–4999	0.109	0.007	0.0	0.118	0.008	0.0	0.077	0.005	0.0
≥5000	0.120	0.011	0.0	0.126	0.012	0.0	0.095	0.011	0.0
Workplace earnings (quartile)									
Lowest	Reference			Reference			Reference		
2^nd^	-0.004	0.005	0.43	-0.003	0.005	0.56	-0.004	0.003	0.29
3^rd^	0.017	0.005	0.0	0.013	0.005	0.02	0.007	0.004	0.05
Highest	0.009	0.006	0.12	0.003	0.006	0.62	-0.000	0.004	0.97
Percentage woman (quartile)									
Lowest	Reference			Reference			Reference		
2^nd^	0.005	0.005	0.31	0.000	0.005	0.93	-0.002	0.004	0.58
3^rd^	0.006	0.006	0.33	-0.002	0.006	0.73	0.003	0.004	0.53
Highest	-0.019	0.007	0.01	-0.026	0.007	0.0	-0.008	0.005	0.10
% fixed contract (quartile)									
Lowest	Reference			Reference			Reference		
2^nd^	0.020	0.005	0.0	0.018	0.005	0.0	0.002	0.003	0.49
3^rd^	0.016	0.005	0.0	0.015	0.005	0.0	0.010	0.004	0.0
Highest	0.006	0.005	0.24	0.005	0.005	0.32	0.002	0.003	0.59
Percentage age>55 (quartile)									
Lowest	Reference			Reference			Reference		
2^nd^	0.018	0.005	0.0	0.017	0.005	0.0	0.000	0.003	0.90
3^rd^	0.019	0.005	0.0	0.019	0.005	0.0	0.004	0.004	0.25
Highest	0.018	0.005	0.0	0.017	0.005	0.0	0.002	0.004	0.59
Coworker averages									
Continued employment									
Employment contract				0.166	0.006	0.0	0.167	0.005	0.0
No employment-contract							-0.034	0.007	0.0
Mental work incapacity				0.288	0.046	0.0	0.128	0.032	0.0
Physical work incapacity				0.058	0.024	0.02	0.017	0.017	0.31
Constant	0.482	0.014	0.0	0.365	0.016	0.0	0.375	0.011	0.0

a The estimated coefficients of the employee characteristics are presented in “Table S1,” in the supplementary material.

Column 2 of table 2 extends the linear probability model with a proxy measure for the extent of disability-related policies and practices within employers (model 2). We found a significant positive association with continuous employment (β=0.166, 95% CI 0.153–0.179). This implies that an increase in this fraction of continued employment of coworkers by 10% increases the probability of continued employment of an individual worker with 1.7%. In the analysis, we accounted for the degree of work incapacities of coworkers assessed in the same year, with 1=most severe work incapacities and 0=least severe work incapacities. Having disabled coworkers with the most severe mental work incapacities increases an employer’s own probability of continued employment by 28.8%. Having disabled coworkers with the most severe physical work incapacities increases an employer’s own probability of continued employment by 5.8%. Supplementary table S1 shows that a larger degree of mental or physical work incapacity of the employee themselves implies a lower probability of continued employment; those with the most severe physical work incapacity have a 58% lower probability of continued employment than those with the least severe work incapacity. For mental work incapacity, we find an even more negative association.

### Variance analysis

Based on the estimated linear probability models, table 3 reports how much of the total variation in employment outcomes was accounted for by employer- and employee characteristics (see table 2 for the corresponding values of regression coefficients of the employer-characteristics and table S1 for the employee-characteristics). Of the explained variance in model 1 (which equaled 19.9%), almost one third (29.0%) was attributed to measured employer characteristics (firm size, sector, and workforce composition). In comparison, almost two-thirds (66.8%) was attributed to included employee characteristics. The remaining part was captured by year-effects (4.2%). Zooming into the set of measured employer characteristics, we see that the sector of employment explained 15.4% of the explained variance, firm size explained 6.0% of the explained variance, and variables related to the composition of a company explained 7.6% of the explained variance.

**Table 3:** Variance analysis of continued employment four months after the assessment (N=84 394).

Characteristics	Explained variance (19.9%)	Unexplained variance (81.1%)	Total variance (100%)
		
	Fraction %	Fraction %	Fraction %
Employer	29.0	5.1	9.9
Sector	15.4		
Firm size	6.0		
Workforce-composition	7.6		
Employee	66.8		13.3
Demographic	0.6		
Socioeconomic	27.1		
Disease type and severity	39.1		
Year-effects	4.2		0.84
Total	100		24.0

Column 2 of table 3 shows that the fraction of the unexplained variance (which equaled 81.1%) that can be attributed to the employer effect (reflecting the importance of disability-related policies and practices) was 5.1% (see Appendix B for the derivation). Taken together, employer characteristics thus explained 9.9% of the total variance in continued employment. Measured employer characteristics related to the type of company explained 5.8% of the total variance (0.199×29.0%), and the employer effect 4.1% (0.811×5.1%). In comparison, included employee characteristics explained 13.3% of the variance (0.199×66.8%).

Next, we evaluated whether the employer effect differs across types of employers (sector and firm size) and employees’ types of diseases. Similar to the above analysis, table 4 presents the fraction of the unexplained variance that can be attributed to the employer effect for different subgroups. We observed differences in the importance of the employer effect between most sectors of employment ranging from 2.7–6.4%. For agriculture (2.7%) and the recreation sector (3.2%), the share of the employer effect is the smallest. For firm size, the employer effect is most substantial for the largest firms, with >5000 employees (9.7%), and small firms with <10 employees (5.9%). From the employee’s side, for disease types, the employer effect is more substantial for assessed employees with mental disorders (5.4%) and musculoskeletal disorders (5.7%) and less relevant for those with cancer (3.1%).

**Table 4:** Employer effect as a fraction of the unexplained variance (mean=5.1%), stratified by sector, firm, and disease type (N=84 394).

Sector		Firm size		Disease type	
		
Group	Fraction %	Group (size)	Fraction %	Group	Fraction %
Agriculture	2.7	1–9	5.9	Cancer	3.1
Recreation	3.2	10–49	1.5	Nervous	4.2
Support services	4.5	50–99	3.7	Circulatory	4.5
Health	4.7	100–249	4.8	Injury	5.0
Manufacturing	4.9	250–499	3.8	Mental	5.4
Public administration	5.1	500–999	5.3	Musculoskeletal	5.7
Education	5.1	1000–4999	5.9		
Wholesale & retail	5.3	≥5000	9.7		
Professional activities	5.5				
Construction	6.2				
Transportation	6.3				
Financial & insurance	6.4				

Finally, we performed the sensitivity analysis that also includes employees with residual work capacity that had no employment contract before the disability assessment (table 2, model 3). We found a near-zero association (β= -0.034, 95% CI -0.048– -0.002) for this group of employees, suggesting that disability-related policies and practices do not contribute to continued employment of workers without an employment contract.

## Discussion

We found that employer characteristics accounted for about 10% of the variability in continued employment of employees with residual work capacity after two years of sick leave. About 6% can be attributed to characteristics related to the type of employer (sector, firm size, and workforce composition) and 4% to an employer effect reflecting the extent of implemented disability-related policies and practices within employers. In comparison, employees’ socio-demographic and disease-related characteristics accounted for about 13% of the variability.

The importance of employer characteristics for continued employment is in line with existing evidence (21). While the prevalence of continued employment was higher among certain types of employers in terms of sector, firm size, and workforce composition, we also found sizeable differences between employers within sectors and firm size groups. These differences may be attributed to differences in implemented disability-related policies and practices. In relative terms, disability-related policies and practices accounted for about forty percent of the total contribution of the employer to continued employment. The total contribution of the employer to continued employment was about as large as employees’ sociodemographic and disease characteristics, which have already been shown to be important factors in previous studies (33). Our results thus indicate that employer characteristics contribute to continued employment of employees with residual work capacity four months after the disability claim assessment.

When considering differences in our findings across employer groups, two findings stand out. First, the prevalence of continued employment was, in general, higher in larger firms than in smaller firms, which has also been found in numerous other studies (6, 21, 26–28). This may suggest that larger firms have more flexibility to accommodate employees with disabilities. We also found that the employer effect was more substantial among larger firms, indicating that not all large firms contribute to continued employment to the same extent through its disability-related policies and practices. Second, the prospect of continued employment of employees with disabilities was particularly low in the construction and low-wage (support) service-orientated sectors. For the construction sector, this finding may stem from higher physical requirements that render job modifications less feasible. Also, most low-wage jobs in service-orientated sectors consist of routine tasks that may limit the ability for employers to offer modified or alternate jobs. Alongside these results, we did not find important differences in the size of the employer effect between most sectors of employment. It was only for the agricultural sector and the recreation sector, sectors with a high share of flexible work arrangements (33), that employers contributed less to continued employment.

Finally, we considered differences in the contribution of employer effects across groups of employees with different chronic diseases. While employees with mental or musculoskeletal disorders have a much lower likelihood of continued employment than employees with cancer, employer effects were also more substantial for these employees. A possible explanation is that employers pay more attention to workplace interventions that assist return to work of employees with mental or musculoskeletal disorders because of their large prevalence and the proven effectiveness of interventions for these two conditions (34, 35). For employees diagnosed with cancer, there may be less scope for improvement since the return to work of cancer survivors after two years of sick leave was already high (68%) compared to those with mental or musculoskeletal disorders (44–45%), as reported in table 1.

### Strengths and limitations

The strength of this study is the large and heterogeneous study population. We included all employees with residual work capacity that applied for long-term work disability benefits. Our study covered all types of diagnosis assessed by an insurance physician and all sectors of employment. By using register data on disability assessments and employment outcomes, we ruled out biases due to selective loss to follow up or misreporting of the degree of disability that is common in cohort studies. Moreover, the setup of the data allowed us to identify coworkers with disabilities to capture both the importance of employer characteristics – as directly measured in the data – and an employer effect that proxies employer-specific disability-related policies and practices affecting all employees within the same employer.

Inferring the importance of disability-related policies and practices from the average employment outcomes of all assessed employees within the same employer provided us with an objective measure not affected by the judgment of employers themselves. Our proxy measure of disability-related policies and practices is in line with the definition by Habeck & Laey (1991). According to them, disability-related policies and practices can be described as a proactive, employer-based approach developed to foster coordinated administrative and rehabilitative strategies to promote cost-effective restoration and return to work (36). Like us, they find that disability-related policies and practices are relatively important for work outcomes, as this includes a wide range of aspects. They refer to the following aspects: top management commitment and supportive policies, coordinated and effective job placement, early intervention and ongoing monitoring, systematic procedures for rehabilitation services, and organized work accommodation.

A possible limitation of our approach is that firm-related factors other than disability-related policies and practices (and the type of organization, ie, firm size, sector, and workforce composition) may have influenced the average employment status four months after the disability assessment, which may contaminate our proxy measure of the employer contribution. Also, the proxy measure of the employer contribution should not be contaminated by unmeasured employee characteristics. This assumption requires two conditions to hold (37, 38). First, there should be no sorting of employees that are more-or-less motivated to continue working after the assessment into specific firms. Second, coworkers that were assessed in the same period should not influence each other’s employment outcomes.

We argue that sorting and peer effects are probably small for two reasons. First, in The Netherlands, the two years period of sick leave is strictly regulated; employers and employees must follow certain procedures to foster re-integration before the employee applies for disability benefits. Second, the rich disability claim assessment data allows us to control for a rich set of socio-demographics employee characteristics and the severity and type of work incapacity of both the employee and coworkers. Therefore, contamination by unmeasured employee or coworker characteristics is likely negligible. The assumption of no contamination by unmeasured employee characteristics is also confirmed if we conduct sensitivity analyses among workers with disabilities with no employment contract before the disability assessment, for whom the employer is unlikely to affect later employment outcomes at the onset of sickness. In line with expectations, an employer effect was non-existent for this group, providing suggestive evidence that there were no sorting or peer effects.

### Implications

The current study provides new insight into the importance of employer characteristics for continued employment of employees with residual work capacity. These findings are important for policymakers, as they provide grounds for tailored strategies to encourage employers to put more effort into facilitating paid employment of employees with residual work capacity. As the prevalence of continued employment was lowest in smaller firms and construction and low-wage service-orientated sectors, investing in tailored strategies to better support these types of employers may be an effective strategy here. There also seems scope for additional investments in disability-related policies and practices among larger firms, as our findings showed that not all larger employers contribute to continued employment to the same extent through their policies and practices. Encouraging employers to take better measures – whereby smaller firms may need additional (financial) government support in doing so – may result in fewer employees with residual work capacity involuntarily leaving the labor force. Similarly, the large differences among employers in their ability to facilitate continued employment of employees with musculoskeletal and mental disorders suggest that there is room for improvement here.

As employment is an important aspect of social participation, any preventive effort by employers could contribute to keeping individuals with disabilities active in society. This is of utmost importance as the number of workers with chronic diseases is expected to increase due to an aging workforce, workforce shortage, and increasing retirement age. In future research, combining register data with survey data about *how* employers have implemented disability-related policies and practices could provide more insight into the content of different kinds of disability-related policies and practices to facilitate continued employment. In addition, more research is needed to examine whether disability-related policies and practices facilitate sustained employment of disabled employees by considering a longer follow-up period.

### Concluding remarks

This study provides support for the potential contribution of employers in supporting employees with residual work capacity due to long-standing health conditions to continue in paid employment. While there are important differences between certain types of employers in terms of sector and firm size, we also found sizeable differences between employers within sectors and firm size groups, which may be attributed to differences in implemented disability-related policies and practices.

## Supplementary material

Supplementary material
