# Supplementary material for: The contribution of employer characteristics to continued employment of employees with residual work capacity: evidence from register data in The Netherlands
Source: Scand J Work Environ Health. 2021 Aug 31;47(6):435–45. doi: 10.5271/sjweh.3961 (PMC8504544; doi:10.5271/sjweh.3961)
Supplement: Supplementary material [file SJWEH-47-435-S001.pdf]

The contribution of employer characteristics to continued employment of employees with residual work capacity: evidence from register data in The Netherlands[1]

by Raun van Ooijen, PhD,2 Pierre WC Koning, PhD, Cécile RL Boot, PhD, Sandra Brouwer, PhD

1. *Supplementary material*
2. *Correspondence to: Raun van Ooijen, University of Groningen, University Medical Center Groningen, Department of Health Sciences, PO-box 30001, 9700 RB Groningen, The Netherlands. [E-mail: r.van.ooijen@umcg.nl]*

Appendix A. Description included employee characteristics

Included socio-demographic variables were age, gender, educational attainment, and earnings. Age was categorized into eight groups (18-29 30-34, 35-39, 40-44, 45-49, 50-54, 55-59, and 60-64 years). Educational attainment was classified into four groups according to the International Standard Classification of Education (ISCED 2011): tertiary (bachelor's or higher), upper secondary (pre-university, general secondary, or senior secondary vocational education), lower secondary (pre-vocational secondary education), and primary education, including persons who did not complete any education. For administrative reasons, educational attainment was missing for 12.6 percent of the sample. We used earnings four months before the disability assessment when all persons in the sample were still employed. Earnings are measured as the hourly wage rate and were categorized into quartiles.

Included disease-related variables were the primary diagnosis, comorbidities, and the degree of mental and physical work incapacity. Primary diagnosis was based on diagnosis codes from the International Classification of Diseases, Tenth Revision (ICD-10) by the World Health Organization (WHO, 1990). We distinguished between the six most prevalent disease categories: mental disorders, musculoskeletal disorders, cancers, circulatory disorders, neurological disorders, and other causes of disability (Chapters II, V, VI, IX, XIII, and all other chapters of ICD-10). Having comorbidities was defined as the presence of either one or two diseases in a different disease category (ICD-10 chapter) co-occurring with the primary diagnosis. The degree of work incapacity was derived from the Functional Ability List, an instrument of 106 items used by the insurance physician to assess the functional limitations of employees applying for disability benefits (39). After performing factor analysis, we derived three work incapacity variables. These variables relate to mental, physical, and autonomously functioning in line with Broersen et al. (39). Since the variable related to autonomously functioning showed limited variability for our sample of employees with residual work capacity, we did not include this variable in the analysis. The derived work incapacity variables were normalized between zero and one: $X_n = (X - X_{min})/(X_{max} - X_{min})$, with one having the most severe work incapacity and zero the least severe work incapacity.

Appendix B. Deriving the importance of the employer-effect for continued employment

Suppose we have a model with only employer characteristic (or 'employer-effects') and thus abstract from employee-related characteristics (or 'employee-effects'):

$$Y_{ikt} = \alpha + w_{kt} + \varepsilon_{ikt},$$

where Y is (continued) employment of individual i at employer k in period t, w is an employer effect, and ε is a residual which may include employee effects. The residual may be correlated between employees within the same employer such that $Cov(\varepsilon_{ikt}, \varepsilon_{jkt}) = \rho\, \sigma_\epsilon^2$, with ρ being the correlation coefficient. We can derive the average employment Y of all employees at employer k, excluding worker i as:

$$\bar{Y}_{kt|i} = \alpha + w_{kt} + \frac{\sum_{-i} \varepsilon_{ikt}}{N_{kt} - 1}.$$

Thus, the covariance between the employment outcome of an employee and the average employment outcome of its coworkers is:

$$Cov(Y_{ikt}, \bar{Y}_{kt|i}) = \sigma_w^2 + Cov\left(\varepsilon_{ikt}, \frac{\sum_{-i} \varepsilon_{jkt}}{N_{kt}-1}\right) = \sigma_w^2 + \frac{\rho\, \sigma_\epsilon^2 (N_{kt}-1)}{N_{kt}-1} = \sigma_w^2 + \rho\, \sigma_\epsilon^2.$$

The variance of a coworker's average employment outcomes is:

$$Var(\bar{Y}_{kt|i}) = Var\left(\alpha + w_{kt} + \frac{\sum_{-i} \varepsilon_{ikt}}{N_{kt} - 1}\right) = \sigma_w^2 + \frac{1}{(N_{kt} - 1)^2} Var\left(\sum_{-i} \varepsilon_{ikt}\right)$$
$$= \sigma_w^2 + \sigma_\varepsilon^2 (N_{kt} - 1)^{-1} (1 + \rho(N_{kt} - 2)).$$

Consequently, the regression coefficient of $\bar{Y}_{jt|i}$ on Y_{ijt} equals:

$$b = \frac{Cov\,(Y_{ikt}, \bar{Y}_{kt|i})}{Var\,(\bar{Y}_{kt|i})} = \frac{\sigma_w^2 + \rho\, \sigma_\varepsilon^2}{\sigma_w^2 + \frac{\sigma_\varepsilon^2 (1 + \rho(N_{kt} - 2))}{N_{kt} - 1}} = \frac{\frac{\sigma_w^2}{\sigma_\varepsilon^2} + \rho}{\frac{\sigma_w^2}{\sigma_\varepsilon^2} + \frac{1 + \rho(N_{kt} - 2)}{N_{kt} - 1}}$$
$$= \frac{(\frac{\sigma_w^2}{\sigma_\varepsilon^2} + \rho)(N_{kt} - 1)}{\frac{\sigma_w^2}{\sigma_\varepsilon^2}(N_{kt} - 1) + 1 + \rho(N_{kt} - 2)}.$$

When ρ= 0 we have:

$$\mathrm{b} = \frac{\eta(N_{kt} - 1)}{\eta(N_{kt} - 1) + 1},$$

with $\eta = \frac{\sigma_w^2}{\sigma_\varepsilon^2}$ is the relative contribution of the employer effect.

The above equation makes apparent that the estimated association between employment outcomes of an employee and the average employment outcome of its coworkers b is determined by (1) the relative contribution of the employer effect η and (2) also the number of assessed coworkers N_{kt}. We take this into account in the analysis.

To give an example of how we derive the employer effect: for firms with two assessed workers, we get $b = \frac{\eta}{\eta+1} \rightarrow \eta = \frac{b}{b-1}$. Based on model 2, we estimate β = 0.097, 95% CI 0.079– 0.115 for firms with two workers. We therefore get an employer effect of η = 0.108, 95% CI 0.086– 0.130. We aggregate the derived employer effects for firms with the same number of assessed coworkers in the same year N_{kt} using inverse-variance weighting, providing us an overall estimate of 0.051 (5.1%) as reported in Table 3.

Table S1. Multiple linear regression analysis with continued employment four months after the assessment as dependent variable [B=coefficients; SE=standard errors; P=P-values].

	Model 1 (N=84,394)			Model 2 (N=84,394)			Model 3 (N=134,101)		
	R^2=0.199			R^2=0.205			R^2=0.303		
Employee characteristics	B	SE	P	B	SE	P	B	SE	P
Age									
18-29	0.034	0.009	0.0	0.032	0.009	0.0	0.079	0.005	0.0
30-34	0.000	0.008	0.98	0.001	0.008	0.92	0.030	0.005	0.0
35-39	-0.004	0.007	0.54	-0.005	0.007	0.49	0.019	0.005	0.0
40-44	0.003	0.007	0.63	0.003	0.007	0.63	0.022	0.005	0.0
45-49	0.008	0.006	0.19	0.008	0.006	0.21	0.022	0.005	0.0
50-54	0.015	0.006	0.01	0.014	0.006	0.02	0.024	0.004	0.0
55-59	0.016	0.006	0.0	0.016	0.006	0.0	0.023	0.004	0.0
60-64	Reference			Reference			Reference		
Gender									
Men	Reference			Reference			Reference		
Women	0.013	0.004	0.0	0.013	0.004	0.0	0.007	0.003	0.02
Education									
Primary	Reference			Reference			Reference		
Lower secondary	0.049	0.005	0.0	0.048	0.005	0.0	0.033	0.003	0.0
Upper secondary	0.095	0.005	0.0	0.091	0.005	0.0	0.060	0.003	0.0
Tertiary	0.103	0.007	0.0	0.098	0.007	0.0	0.065	0.005	0.0
Missing	0.249	0.006	0.0	0.243	0.006	0.0	0.178	0.004	0.0
Earnings									
lowest quartile	Reference			Reference			Reference		
2nd quartile	0.064	0.005	0.0	0.063	0.005	0.0	0.063	0.004	0.0
3rd quartile	0.128	0.005	0.0	0.126	0.005	0.0	0.132	0.004	0.0
highest quartile	0.189	0.006	0.0	0.185	0.006	0.0	0.214	0.005	0.0
Primary diagnosis									
Cancer	0.142	0.006	0.0	0.139	0.006	0.0	0.118	0.005	0.0
Mental	-0.036	0.005	0.0	-0.037	0.005	0.0	-0.030	0.004	0.0
Nervous	0.099	0.008	0.0	0.098	0.008	0.0	0.059	0.006	0.0
Circulatory	0.110	0.007	0.0	0.110	0.007	0.0	0.074	0.005	0.0
Musculoskeletal	Reference			Reference			Reference		
Injury	0.056	0.006	0.0	0.057	0.006	0.0	0.038	0.004	0.0
Other	0.041	0.005	0.0	0.040	0.005	0.0	0.022	0.004	0.0
Comorbidities									
No	Reference			Reference			Reference		
Yes	-0.043	0.003	0.0	-0.042	0.003	0.0	-0.031	0.002	0.0
Work incapacity									
Mental	-1.433	0.025	0.0	-1.435	0.025	0.0	-1.066	0.018	0.0
Physical	-0.581	0.014	0.0	-0.581	0.014	0.0	-0.471	0.010	0.0

All models also include year effects.
